# Safety of COVID-19 Vaccine in Patients with Rheumatic and Musculoskeletal Diseases

**DOI:** 10.31138/mjr.140223.sof

**Published:** 2024-02-08

**Authors:** Ali Abdulrahman Younis, Asal Adnan Ridha, Yasameen Abbas Humadi, Nizar Abdulateef Jassim, Nabaa Ihsan Awadh, Avin Maroof, Ali Mohammed Hussein Alqazzaz, Faiq I. Gorial, Taha Ahmed Qaradaghi, Zahraa Salam Abdulzahra, Zainab A. Mahmood, Noor Talal Yaseen, Duaa Nidhal AlIdrecy, Israa Talib Hakman, Saja Jabbar Tarfah, Adil Saudi Khudhair

**Affiliations:** 1 Department of Medicine, College of Medicine, University of Mosul, Mosul, Iraq,; 2 Rheumatology Unit, Department of Internal Medicine, Baghdad Teaching Hospital, Medical City, Iraq,; 3 Al-Nahrain University, College of Medicine, Baghdad, Iraq,; 4 Rheumatology Unit, Department of Medicine, College of Medicine, University of Baghdad, Iraq,; 5 School of Medicine, University of Kurdistan-Hawler, Erbil, Kurdistan Region, Iraq,; 6 Department of Medicine, College of Medicine, University of Babylon, Iraq,; 7 Sulaymaniyah Internal Medicine Teaching Hospital, Sulaymaniyah, Iraq,; 8 College of Medicine, University of Basrah, Basrah, Iraq,; 9 Al-Imamain Al-Kadhimin Medical City, Baghdad, Iraq,; 10 Baghdad Teaching Hospital, Baghdad, Iraq

**Keywords:** COVID-19, vaccine, safety, rheumatic diseases, flare

## Abstract

**Objectives:**

The main purpose of this study was to determine the frequency of COVID-19 vaccine side effects in patients with rheumatic diseases and to examine any potential associations with medications, disease type, or comorbidities.

**Methods:**

A multicentre cross-sectional study from rheumatology units in different hospitals in Iraq was carried out between 8^th^ of August 2021 and 4th of August 2022. Patients were eligible for inclusion if they have a rheumatic disease and have taken one or more doses of any COVID-19 vaccine.

**Results:**

A total of 661 (57.8% female, mean age 46.51± 12.97 years) patients with rheumatic illnesses who received the “COVID-19” vaccination were included in this study. Rheumatoid arthritis was the most frequent diagnostic group. The Pfizer vaccine was given to the majority of patients (74.6%), followed by Sinopharm (16.2%), and AstraZeneca (9.2%). Side effects were detected in 661(100%) and 528 (100%) patients following the first and second vaccination doses, respectively; among which the most frequent were injection site pain in 57.8% following the first dose and 47.6% after the second dose, followed by fatigue and fever. According to multivariate logistic regression models, age (B=−0.204, p = 0.000), had a significantly inverse correlation coefficient with the experience of greater side effects. Rheumatic disease flares reported in 9.9%, 10.3%, and 8.2% of patients who received the Pfizer, Sinopharm, and AstraZeneca vaccines, respectively.

**Conclusion:**

The “COVID-19” vaccination has a reassuring safety profile with no greater risk of adverse events in any specific illness or pharmacological therapy.

## INTRODUCTION

Since December 2019, the “severe acute respiratory syndrome coronavirus 2 (SARS-CoV-2)” has emerged as a serious global health emergency, infecting over 81 million people and killing over one million, causing global health and economic challenges and forcing the entire world to follow strict isolation protocols.^1^

Many observational studies have segregated risk factors for mortality in COVID-19 infection and consistently, immunosuppression stands out as a well-identified risk. Increased mortality was found in patients with lupus, rheumatoid arthritis (RA), and psoriatic arthritis, compared to those having no rheumatic diseases with an adjusted odds ratio [aOR] of 1.30. In addition, patients with rheumatic disorders were more likely to be hospitalised, especially those receiving large doses of glucocorticoids (≥10 mg/day), with an odd ratio of 2.09.^2^ It has been also demonstrated that mycophenolate mofetil and rituximab are significantly linked to worse outcomes after SARSCoV-2 infection. ^3^

Vaccination is efficacious in limiting transmission, morbidity, and mortality. However, the COVID-19 vaccination is infamous due to the combined causes of public misinformation, post-vaccination reports of adverse effects, and scepticism related to the short production period. ^4^

It is critical to address anti-vaccine concerns in order to improve vaccination rates and achieve protection for patients with rheumatic disease who are hesitant to receive vaccination due to side effect concerns.^5^

Four vaccines were registered in Iraq, but only three were eventually purchased by the government, namely: Pfizer/BioNTech, Gamaleya, Oxford/AstraZeneca Vaxzevria, and Sinopharm (Beijing).^6^ As there is a lack of information addressing patient fears and vaccination adverse events in Iraqi patients with rheumatic diseases, this study aimed to gather information regarding the frequency of COVID-19 vaccine side effects in Iraqi patients with rheumatic diseases and to look for any possible relationships with drugs, disease type, or comorbid illnesses.

## MATERIALS AND METHODS

### Study design and settings

A multicentre, “cross-sectional” observational study was carried out at the rheumatology clinics in 7 tertiary hospitals providing care for patients with rheumatic diseases in Iraq during the period from August 8^th^, 2021, until August 4^th^, 2022. Consecutive patients receiving follow-up care in these rheumatology clinics were recruited. Data was collected by the practicing physicians and postgraduate Rheumatology students through structured interview conducted face-to-face.

Generally speaking, patients with rheumatic diseases routinely underwent a face-to-face evaluation before receiving the COVID vaccine. Patients were asked to report any adverse event either via telephone or by attending to the clinic. Patients with suspected disease reactivation underwent a face-to-face evaluation in order to confirm the flare and record the associated characteristics.

### Participants

Any patient with mechanical or inflammatory rheumatic disease aged ≥18 years who received at least one dose of any of the three available COVID vaccines was included in the study. No patient had received a booster dose during the study period.

### Data collection

A questionnaire was constructed to gather the following data through direct interview, in addition to the information available in patients records: Age, gender, disease type, disease activity state at the time of immunisation, drugs used at the time of vaccination, use and dose of steroids, if any, and history of COVID infection prior to vaccination. Data regarding the type of vaccine, number of doses, COVID infection after vaccination, and if positive, the duration between vaccination and infection, and the sequelae of infection; disease status after vaccination; any adverse events after receiving the vaccine, and the need for medical interference for the adverse events, if any were also included.

### Statistical Analysis

The information was extracted to an Excel sheet and managed by the Statistical Package for Social Sciences version 26 SPSS 26 software. Continuous variables were expressed in mean±standard deviation (SD). Categorical variables were expressed as numbers and percentages; the duration was expressed in median and range. To assess possible predictors of adverse reaction to vaccination, multivariate logistic regression analysis was used with a statistical significance set to a p-value of less than 0.05.

## RESULTS

A total of 661 patients with rheumatic disease who received the COVID vaccination were included in the study. Of those, 382 (57.8%) were female, the mean age was 46.51± (SD 12.97), and the 40–60 age group is the most frequent group representing 48.0%. Except for those aged 20 to 40, women outnumbered men in all age groups. Only 29 people in the study had co-morbidities, with hypertension (17, 2.6%), diabetes (7, 1.1%), and IHD (3, 0.5%) being the most common (**Table 1**).

**Table 1.** Baseline demographic and clinical characteristics of the study sample.

**Age group intervals**	**Gender**		**Total No. (%)**
	**Males No. (%)**	**Females No. (%)**	
**<20 years**	1(0.4)	5(1.3)	6(0.9)
**20–40 years**	112(40.1)	96(25.1)	208(31.5)
**40–60 years**	126(45.2)	191(50.0)	317(48.0)
**≥60years**	40(14.3)	90(23.6)	130(19.7)
**Total**	279	382	661
**Mean age /years**	46.51±12.97		
**Co-morbidities**	**Frequency**		**Percentage**
**HT**	17		2.6
**DM**	7		1.1
**IHD**	3		0.5
**Hypothyroid**	1		0.2
**Stroke**	1		0.2
**Disease Activity at time of vaccination**			
Remission or low disease activity	563		85.2
Moderate disease activity	93		14.1
High disease activity	5		0.7
**Frequency of COVID-19 infection before vaccination**			
Yes	275		41.6
No	386		58.4
**Diseases**			
Rheumatoid arthritis	202		30.6
Non inflammatory/mechanical 34(4): RMDs	156		23.6
Axial SpA	146		22.1
SLE	39		5.9
PsA	34		5.1
Behcet disease	20		3.0
Vasculitis	18		2.7
Scleroderma	10		1.5
Dermatomyositis or polymyositis	9		1.4
Overlap syndrome	6		0.9
Sjögren’s syndrome	5		0.8
Enteropathic arthritis	5		0.8
Reactive arthritis	4		0.6
Adult onset Still’s disease	3		0.5
Gout	2		0.3
Fibromyalgia	1		0.1
Relapsing polychondritis	1		0.1

**Drug exposure**	**Frequency**	**Percentage**
TNF-α inhibitors	265	40.1
Methotrexate	174	26.3
NSAIDs	130	19.7
Steroid	115	17.4
Azathioprine	76	11.5
Hydroxychloroquine	64	9.7
Rituximab	53	8.0
Leflunomide	31	4.7
Mycophenolate	18	2.7
Colchicine	9	1.4
Sulfasalazine	7	1.1
Aspirin	4	0.6
Cyclophosphamide	5	0.8
Cyclosporine	4	0.6

TNF-α: tumour necrosis factor alpha; NSAIDs: non-steroidal anti-inflammatory drugs; RMDs: rheumatic and musculoskeletal diseases; SpA: spondyloarthritis; SLE: systemic lupus erythematosus; PsA: psoriatic arthritis.

Tumour necrosis factor alpha (TNF- α), inhibitors (40.1%), methotrexate (26.3%), and non-steroidal anti-inflammatory drugs (NSAIDs) (19.7%) were the most commonly used medications, as shown in **Table 1**. Among the study sample, 41.6% have COVID-19 infection before the vaccination, as shown in **Table 1**.

Concerning the type of rheumatic diseases among the vaccinated patients, rheumatoid arthritis was the most prevalent rheumatic disease (30.6%), followed by non-inflammatory/mechanical rheumatic and musculoskeletal diseases (RMDs) (23.6%) and Axial spondyloarthritis (SpA) (22.1%), while the other types represent 23.7% as shown in **Table 1**. The majority of the patients (85.2%) were in remission or low disease activity at the time of vaccination (**Table 1**).

**Table 2** demonstrates the types of COVID-19 vaccine received among rheumatic disease patients and shows that 493 (74.6%) out of the sample took Pfizer vaccine; 294 (44.5%) were females and 199 (30.1%) were males; most of them received 2 doses. Sinopharm vaccine had been taken by 107 (16.2%) of the study sample; the majority of them received 2 doses. AstraZeneca vaccine had been taken by 61 (9.2%) patients; most of them received 2 doses of vaccine.

**Table 2.** Types of COVID-19 vaccine received among rheumatic disease patients.

**Types of COVID-19 vaccinations**		**One dose (n=133) No. (%)**	**Two doses (n=528) No. (%)**	**Total No. (%)**
**Pfizer**	**Males**	42(31.6)	157(29.7)	199(30.1)
	**Females**	62(46.6)	232(43.9)	294(44.5)
**Sinopharm**	**Males**	13(9.8)	38(7.3)	51(7.7)
	**Females**	10(7.5)	46(8.7)	56(8.5)
**AstraZeneca**	**Males**	2(1.5)	27(5.1)	29(4.4)
	**Females**	4(3.0)	28(5.3)	32(4.8)

**Table 3** demonstrates the COVID-19 infection after vaccination and shows that the COVID-19 infection occurred in 8 patients after the first dose; 6 out of the 8 were after the Pfizer vaccine and 2 were found after the Sinopharm vaccine; and 22 patients after the second dose; 14 after the Pfizer vaccine, 4 after the Sinopharm vaccine, and 4 after the AstraZeneca vaccine. The median duration of infection after the first Pfizer dose is 9 days, and it is 50 days after receiving the second dose. After the “Sinopharm vaccine”, the median duration is 10.5 days after receiving the first dose and 45 days following the second dose. No COVID-19 infection was found after the first dose of AstraZeneca; only 4 patients got COVID-19 after the second dose, with a median duration of 37 days.

**Table 3.** COVID-19 infection after vaccination.

		**First dose No. (%)**	**Second dose No. (%)**
**COVID-19 diagnosis after each type of vaccination**	**Pfizer**	6(75.0)	14(63.6)
	**Sinopharm**	2(25.0)	4(18.2)
	**AstraZeneca**	0(0.0)	4(18.2)
		**Median(range)**	**Median(range)**
**Days after vaccination infection with COVID had occurred**	**Pfizer**	9(3–15)	50(1–190)
	**Sinopharm**	10.50(7–118)	45(14–60)
	**AstraZeneca**	0(0)	37(14–80)

Regarding the medical management of COVID-19 after vaccination, 23 patients were treated at home, two patients needed hospitalisation with standard care but no oxygen, three patients required oxygen after hospitalisation, and only one patient needed admission to the ICU. **Table 4** shows types of rheumatic diseases flare among vaccinated patients and reveals that 49 (9.9%) patients of those received Pfizer vaccine reported flaring of the disease after vaccination with a median interval of 5 days and the most frequent flare types were arthritis 27(5.5%), arthralgia 23(4.7%), and fatigue 12(2.4%). Concerning the Sinopharm vaccine, the flaring up occurs in 11(10.3%) with a median interval of 4 days and the most frequent types were arthralgia 7(6.5%), arthritis 3(2.8%), and cutaneous flare 2(1.9%). Regarding the AstraZeneca vaccine, the flare developed in 6 patients (8.2%) with a median interval of 7.5 days and was in the form of arthralgia 4(6.6%), fatigue 2(3.3%), and arthritis 1(1.6%). The difference among the study groups regarding the disease flare after the vaccinations is statistically insignificant (p=0.993). All the flares occurred in patients with inflammatory rheumatic diseases.

**Table 4.** Type of rheumatic diseases flare among vaccinated patients.

**Flare**	**Disease flare after vaccination**		**Days after vaccination disease flare had occurred**	**Frequency of Flare types**					
	**Yes No. (%)**	**No No. (%)**		**Types**	**Frequency**
**Pfizer Median (range)**	49(9.9)	444(90.1)	5(1–30)	Arthritis	27(5.5)
				Arthralgia	23(4.7)
				Fatigue	13(2.6)
				Cutaneous flare	6(1.2)
				Neuro-psychiatric	1(0.2)
				Mouth ulcer	2(0.4)
				LN enlargements	1(0.2)
				Fever	2(0.4)
				Renal	1(0.2)
				Backache	2(0.4)
				Arthralgia	7(6.5)
**Sinopharm Median (range)**	11(10.3)	96(89.7)	4(1–20)	Arthritis	3(2.8)
				Cutaneous flare	2(1.9)
				Fatigue	2(1.9)
				Arthralgia	4(6.6)
				Fatigue	2(3.3)
**AstraZeneca Median (range)**	6(8.2)	55(91.8)	7.5(2–30)	Arthritis	1(1.6)
				Muscle weakness	1(1.6)
				Neuro-psychiatric	1(1.6)

LN: lymph node.

Side effects were detected in 661(100%) and 528 (100%) patients following the first and second vaccination doses, respectively. Most of the side effects for all COVID-19 vaccines included in the study, whether the 1^st^ or 2nd dose, developed within day zero and subsequent days 1 and 2, as shown in **Table 5**.

**Table 5.** Timing of onset of side-effects after COVID-19 vaccines, days.

**Timing of onset of side-effects /days**	**Total No. (%)**	
	1^**st**^ **dose**	2^**nd**^ **dose**
≤ 24 hr	328(49.6)	223(42.2)
Day 1	211(31.9)	239(45.3)
Day 2	86(13)	46(8.7)
Day 3	33(5)	14(2.7)
Day 4	1(0.2)	2(0.4)
Day 5	1(0.2)	4(0.8)
Day 9	1(0.2)	0(0.0)
Total	661	528

**Table 6** demonstrates the side effects experienced from COVID-19 vaccine and shows that the most frequently detected side effect was pain at site of injection which developed in 382(57.8%) after the first dose and 253(47.6%) after the second dose, primarily due to the Pfizer vaccine. Fatigue developed in 230(34.8%) after the first dose and 190(35.7%) after the second dose, mostly occurs with AstraZeneca vaccine. Fever occurred in 229(34.6%) after the first dose and 154(28.9%) after the second dose, mostly in Pfizer and AstraZeneca vaccines. **Table 7** demonstrates the requirement for consultations or hospital admissions due to side effects, showing that 2 (0.3 %) patients were admitted to hospital, 29 (4.4%) patients needed medical consultations, and 2 (0.3%) patients needed emergency consultations.

**Table 6.** Side effects experienced from COVID-19 vaccine.

**Side-effects after first and second dose**	**Type of vaccinations**						**Total No. (%)**	
	**Pfizer**		**Sinopharm**		**AstraZeneca**			
	**No. (%)**		**No. (%)**		**No. (%)**			
	1^**st**^ **dose**	2^**nd**^**dose**	1^**st**^ **dose**	2^**nd**^ **dose**	1^**st**^ **dose**	2^**nd**^ **dose**	1^**st**^ **dose**	2^**nd**^ **dose**
Pain at site of injection	307(62.3)	204(52.3)	38(35.5)	24(29.7)	37(60.7)	25(44.6)	382(57.8)	253(47.9)
Fatigue	178(36.1)	154(39.5)	25(23.4)	13(15.1)	27(44.3)	23(41.1)	230(34.8)	190(35.9)
Fever	175(35.5)	133(34.1)	25(23.4)	9(10.5)	29(47.5)	12(21.4)	229(34.6)	154(28.9)
Headache	75(15.2)	0(0.0)	17(15.9)	6(7.0)	15(24.6)	11(19.6)	107(16.2)	17(3.2)
Myalgia	66(13.4)	63(16.2)	12(11.2)	8(9.3)	15(24.6)	9(16.1)	93(14.1)	80(15.2)
Chills	16(3.2)	11(2.8)	1(0.9)	2(2.3)	4(6.6)	1(1.8)	21(3.2)	14(2.6)
Arthralgia	4(0.8)	3(0.8)	0(0.0)	0(0.0)	0(0.0)	0(0.0)	4(0.6)	3(0.6)
Nausea/vomiting	6(1.2)	8(2.1)	2(1.9)	0(0.0)	2(3.3)	0(0.0)	10(1.5)	8(1.5)
Elevated liver enzymes	1(0.2)	0(0.0)	0(0.0)	0(0.0)	0(0.0)	0(0.0)	1(0.2)	0(0.0)
Thrombosis	1(0.2)	0(0.0)	0(0.0)	0(0.0)	0(0.0)	0(0.0)	1(0.2)	0(0.0)
Vasculitis	1(0.2)	0(0.0)	0(0.0)	0(0.0)	0(0.0)	0(0.0)	1(0.2)	0(0.0)
Sore throat	1(0.2)	0(0.0)	0(0.0)	0(0.0)	0(0.0)	0(0.0)	1(0.2)	0(0.0)
Lymphadenopathy	1(0.2)	0(0.0)	0(0.0)	0(0.0)	0(0.0)	0(0.0)	1(0.2)	0(0.0)
Skin rash	0(0.0)	1(0.3)	1(0.9)	0(0.0)	0(0.0)0	0(0.0)	1(0.2)	1(0.2)
Flu like illness	0(0.0)	1(0.3)	1(0.9)	1(1.2)	0(0.0)	0(0.0)	1(0.2)	2(0.4)
Palpitation	0(0.0)	0	2(1.9)	0	0(0.0)	0(0.0)	2(0.3)	0(0.0)
Left shoulder pain	0(0.0)	0(0.0)	1(0.9)	1(1.2)	0(0.0)	0(0.0)	1(0.2)	1(0.2)
Cough	1(0.2)	2(0.5)	0(0.0)	0(0.0)	1(1.6)	0(0.0)	2(0.3)	2(0.4)
Chest pain	0(0.0)	0(0.0)	0(0.0)	0(0.0)	1(1.6)	0(0.0)	1(0.2)	0(0.0)
Dysphagia	0(0.0)	0(0.0)	0(0.0)	0(0.0)	1(1.6)	0(0.0)	1(0.2)	0(0.0)
Muscle weakness	0(0.0)	0(0.0)	0(0.0)	0(0.0)	0(0.0)	1(1.8)	0(0.0)	1(0.2)
**Total**	493	390	107	86	61	56	661	528

**Table 7.** Need for consultations or hospital admission for side-effects.

**Consultations or admissions to hospital for side-effects**	**Type of vaccinations**			**Total No. (%)**
	**Pfizer**	**Sinopharm**	**AstraZeneca**	
	**No. (%)**	**No. (%)**	**No. (%)**	
**Admission to hospital**	1(0.2)	1(0.9)	0(0.0)	2(0.3)
**Medical consultation**	23(4.7)	4(3.8)	2(3.3)	29(4.4)
**Emergency consultation**	1(0.2)	0(0.0)	1(1.6)	2(0.3)
**Non**	468(94.9)	102(95.3)	58(95.1)	628(95.0)
**Total**	493	107	61	661

A multivariate logistic regression analysis examining variables with possible effect on the occurrence of vaccine side effects reveals that age has a statistically significant inverse coefficient (B= −0.204, p <0.001); type of rheumatic diseases, comorbid illnesses, disease activity and medications were not associated with more side effects (**Table 8**).

**Table 8.** Multivariate logistic regression analysis on factors associated with experiencing side effects of the vaccine.

**Model**	**Unstandardised Coefficients**		**Standardised Coefficients**	**p-value**
	**B**	**Std. Error**	**Beta**	
(Constant)	6.224	0.848		0.000
Age	−0.750	0.141	−0.204	<0.001
Gender	−0.266	0.214	−0.048	0.215
Hypertension	−0.101	0.121	−0.036	0.401
Diabetes mellitus	−0.392	0.239	−0.173	0.324
Vaccination	−0.038	0.163	−0.009	0.814
Rheumatoid arthritis	0.592	0.216	0.322	0.068
Non inflammatory/mechanical RMDs	0.543	0.094	0.327	0.059
Axial SpA	0.214	0.101	0.611	0.143
Vasculitis	0.094	0.032	0.021	0.856
SLE	0.153	0.045	0.043	0.766
Disease Activity at time of vaccination	−0.231	0.453	−0.238	0.496
Drug exposure	−0.043	0.022	−0.032	0.231
Steroid	0.674	0.119	0.491	0.623
Steroid dose	0.083	0.084	0.045	0.322
TNF- inhibitors	0.043	0.112	0.074	0.123
Methotrexate	0.327	0.054	0.192	0.871
Azathioprine	0.656	0.210	0.439	0.073
Hydroxychloroquine	0.385	0.134	0.346	0.306
Rituximab	0.270	0.097	0.231	0.198
Leflunomide	0.328	0.178	0.411	0.174
Mycophenolate	0.376	0.044	0.276	0.099
Sulfasalazine	0.402	0.361	0.402	0.219
Cyclophosphamide	0.199	0.472	0.338	0.111
Cyclosporine	0.112	0.365	0.322	0.067

RMDs: rheumatic and musculoskeletal diseases; SpA: spondyloarthritis; SLE: systemic lupus erythematosus.

## DISCUSSION

An increased risk of developing severe COVID-19 infections has been evidenced in rheumatic disease patients, and COVID-19 vaccination is strictly recommended for these patients. Data on the safety and effectiveness of COVID-19 vaccines in people with rheumatic disorders, however, are still inconclusive. An issue that is becoming more crucial is the potential emergence of adverse events, including flare-up of the underlying rheumatic disease.

This study investigated the safety of three different types of “COVID-19” vaccines among Iraqi patients with rheumatic disorders and reported that the SARS-CoV-2 vaccinations’ safety profile in this cohort was reassuring. The vast majority of patients responded favourably to their vaccine, and flare-ups of rheumatic disorders were quite uncommon for all types of vaccines with a median interval of 4–7.5 days post vaccination. The majority of flare manifested as arthritis, arthralgia, and fatigue, with very rare reports of neuro-psychiatric and renal flares (0.2%). Most side effects related to vaccines were mild, involving temporary local and systemic symptoms with only (4.4%, 0.3%, 0.3%) requiring medical consultation, emergency consultation, and hospital admission respectively.

In terms of flares, our data suggested that the risk of rheumatic diseases flare after vaccination is low and not substantially associated with any specific type of vaccine, with the observed percentages being consistent with other observational studies that have documented the flare rate of rheumatic diseases after COVID-19 vaccination, ranging from 0.4% to 20%,^7–28^ and in agreement with the findings of a meta-analysis of these studies that found comparable flare rates of rheumatic disease after “mRNA” vaccination and “ adenovirus”-based vaccination, which were 7% (,5%–9%; P=0.000) and 8% (4%–12%; P=0.000), respectively.^29^ Similar to the results of our study, the majority of flares developed soon after receiving the COVID-19 immunisation and consistently occurred within the first week. Visentini et al. also obtained similar results, as 83% of the flare occurred within seven days.^11^ Concerning flare severity, most of the flares after “COVID-19” vaccination were related mainly to musculoskeletal and cutaneous manifestations, and this finding was confirmed by multiple studies that demonstrated that the flare-up of rheumatic illnesses following COVID-19 immunization primarily manifested as joint pain, stiffness, and swelling, particularly for inflammatory arthritis.^7,8,10,12,17,18,22,23^ Regarding SLE, in addition to arthritis, cutaneous and mucosal manifestations, such as malar erythema and alopecia, were also frequent.^7,11,13,17^ Additionally, fatigue and myalgia are frequently experienced by flare sufferers.^12,16–18^

Furthermore, other studies investigated disease activity before and after immunisation and discovered no discernible difference in the overall disease activity of rheumatic diseases, so indirectly demonstrating an insignificant flare-up.^14,25,30,31^

Regarding vaccination side effects, the frequency and type of side effects were similar between vaccines, with the possible exception of a slightly greater frequency of discomfort at the injection site with the Pfizer vaccine and fatigue with the AstraZeneca vaccine. The adverse effect data are consistent with earlier studies in rheumatic illnesses patients.^12,17,22,32–35^ We underline that thrombocytopenia events were not reported in our study population.

When post vaccination side effects were assessed by multivariate regression analysis for age, rheumatic diseases type, comorbidities, disease activity, and medications; the age was the only variable that showed a statistically significant inverse coefficient. However, a similar result was obtained by Li YK et al.,^36^ with adverse effects were more frequently reported in young patients. Whilst principal objective of our study was to gather safety information about the three COVID-19 vaccines that were available in Iraq among rheumatic diseases patients, we also gathered information regarding breakthrough infections and discovered that these occurred very infrequently, especially in fully vaccinated patients, and were more frequently reported with the Pfizer vaccine.

According to a systematic review and network meta-analysis assessing the clinical effectiveness of COVID-19 vaccines,^32^ which included eight phase 3 randomised controlled trials (Rotshild et al.). mRNA vaccines decreased the risk of symptomatic COVID-19 more than other vaccines, but there was no difference in preventing severe disease.^37^ It should be noted that the studies’ methodology varied greatly and that this was an indirect comparison of the vaccines. Although evaluating the outcome of post vaccination COVID-19 infection was not the primary objective of this study, it was found that 81% were treated at home, two patients required hospitalisation without O2, and three patients required O2, but only one patient needed ICU admission. These findings, however, are insufficient to conclude that one vaccine is more effective than another for protecting against COVID-19 infection in those with rheumatic illnesses. This study was the first to give a descriptive analysis and assessment of contributing factors and adverse effects of the COVID-19 vaccine in Iraqi patients with rheumatic illnesses. The sample size is a significant drawback, and the lack of a control group made it impossible to compare the patient’s symptoms and side effects to those of people with similar conditions or the general population; and patient self-reported symptoms and side effects were at risk of recall bias.

In conclusion, our findings present an encouraging picture regarding the safety of “COVID-19” vaccination in patients with rheumatic disorders. Almost all the reported side effects were mild to moderate. Reassuringly, no serious flare up of underlying rheumatic disorder was documented after vaccination. These data need to be disseminated to reduce vaccine hesitancy in patients with rheumatic diseases.
